# Cellular vimentin regulates the infectivity of Newcastle disease virus through targeting of the HN protein

**DOI:** 10.1186/s13567-023-01230-5

**Published:** 2023-10-17

**Authors:** Xiaolong Lu, Kaituo Liu, Yu Chen, Ruyi Gao, Zenglei Hu, Jiao Hu, Min Gu, Shunlin Hu, Chan Ding, Xinan Jiao, Xiaoquan Wang, Xiufan Liu, Xiaowen Liu

**Affiliations:** 1 Animal Infectious Disease Laboratory, College of Veterinary Medicine, Yangzhou University, No.48 East Wenhui Road, Yangzhou, 225009 China; 2 Jiangsu Key Laboratory of Zoonosis, Yangzhou University, Yangzhou, 225009 China; 3 Jiangsu Co-Innovation Center for Prevention and Control of Important Animal Infectious Diseases and Zoonoses, Yangzhou, 225009 China; 4 Joint International Research Laboratory of Agriculture and Agri-Product Safety, Ministry of Education of China, Yangzhou University, Yangzhou, 225009 China; 5 Department of Avian Infectious Diseases, Shanghai Veterinary Research Institute, Chinese Academy of Agricultural Science, Shanghai, 200000 China

**Keywords:** Newcastle disease virus, HN, vimentin, viral infection, macrophage

## Abstract

The haemagglutinin-neuraminidase (HN) protein plays a crucial role in the infectivity and virulence of Newcastle disease virus (NDV). In a previous study, the mutant HN protein was identified as a crucial virulence factor for the velogenic variant NDV strain JS/7/05/Ch, which evolved from the prototypic vaccine strain Mukteswar. Furthermore, macrophages are the main susceptible target cells of NDV. However, the possible involvement of cellular molecules in viral infectivity remains unclear. Herein, we elucidate the crucial role of vimentin, an intermediate filament protein, in regulating NDV infectivity through targeting of the HN protein. Using LC‒MS/MS mass spectrometry and coimmunoprecipitation assays, we identified vimentin as a host protein that differentially interacted with prototypic and mutant HN proteins. Further analysis revealed that the variant NDV strain induced more significant rearrangement of vimentin fibres compared to the prototypic NDV strain and showed an interdependence between vimentin rearrangement and virus replication. Notably, these mutual influences were pronounced in HD11 chicken macrophages. Moreover, vimentin was required for multiple infection processes of the variant NDV strain in HD11 cells, including viral internalization, fusion, and release, while it was not necessary for those of the prototypic NDV strain. Collectively, these findings underscore the pivotal role of vimentin in NDV infection through targeting of the HN protein, providing novel targets for antiviral treatment strategies for NDV.

**Supplementary Information:**

The online version contains supplementary material available at 10.1186/s13567-023-01230-5.

## Introduction

Newcastle disease virus (NDV) is also termed avian paramyxovirus type 1 (APMV-1) and is a member of the genus Orthoavulavirus and family Paramyxoviridae [1]. NDV strains can be divided into three groups based on their virulence: avirulent (lentogenic), intermediate (mesogenic), and highly virulent (velogenic) [2]. A critical envelope protein, the HN protein has been extensively demonstrated to play an important role in regulating NDV infection and virulence [3, 4]. Close interactions between viral proteins and host proteins are essential for viral infection, and several proteins of NDV have been confirmed to modulate viral infection by interacting with host proteins. For instance, the interaction between NDV NP and eukaryotic initiation factor 4E (eIF4E) contributes to the selective cap-dependent translation of viral mRNA, thereby enhancing viral infection [5]; the interaction between NDV P and the CARD11 protein can inhibit NDV replication by impairing the activity of viral RNA polymerase [6]. Therefore, it is crucial to elucidate the precise mechanism of NDV infection by investigating the interactions between viral and host proteins.

Vimentin, an important type III intermediate filament cytoskeletal protein, has been widely reported to be involved in multiple steps of viral infection [7]. Vimentin plays a key role in the viral adsorption and entry process, which is the initial and crucial step of viral infection. Vimentin can be bound by the spike (S) protein and serve as a cellular coreceptor for SARS-CoV during infection, thereby promoting the adsorption and entry of SARS-CoV [8]. A similar phenomenon is observed during infection with the novel coronavirus (SARS-CoV-2), which caused the coronavirus disease 2019 (COVID-19) pandemic. Vimentin can interact with the S protein of SARS-CoV-2 and act as a key component of the S-ACE2 complex, thus increasing the adsorption and entry of SARS-CoV-2 [9, 10]. Furthermore, vimentin can facilitate the adsorption and entry of other viruses, such as porcine reproductive and respiratory syndrome virus (PRRSV) [11] and Japanese encephalitis virus (JEV) [12]. Moreover, viral internalization and fusion, important steps of infection following viral adsorption, can be regulated by vimentin. For instance, vimentin can inhibit the internalization of human papillomavirus type 16 (HPV16) [13] and the fusion of influenza A virus (IAV) [14]. The assembly and release of viruses play an important role in their spread. Vimentin has been shown to promote the assembly of various viruses, such as frog virus 3 (FV3) [15] and vaccinia virus (VACV) [16]. The destruction of the vimentin filament structure can impede the release of certain viruses, such as bluetongue virus (BTV) [17] and dengue virus type 2 (DENV-2) [18], while in some cases, it may facilitate the release of FMDV [19]. It is noteworthy that vimentin plays a dual role in viral replication, acting as both a facilitator and an inhibitor. Vimentin, a well-known receptor for PRRSV, can interact with annexin A2 (ANXA2) to promote PRRSV replication [20]. In addition, vimentin is essential for the formation of the replication complex of foot-and-mouth disease virus (FMDV), thereby facilitating viral replication [19]. Additionally, vimentin has been shown to have a positive impact on the replication of several other viruses, including Chandipura virus (CHPV) [21], avian influenza virus (AIV) [22] and transmissible gastroenteritis virus (TGEV) [23]. In some cases, however, vimentin has a negative regulatory effect on viral replication. For example, vimentin acts as a direct binding partner of the FMDV 3A protein and negatively regulates FMDV replication [24]; vimentin can also restrict HIV replication by regulating the interaction between the HIV Gag protein and the host M2BP protein [25]. However, the precise role of vimentin in NDV infection has yet to be fully characterized.

We previously isolated the velogenic NDV strain JS/7/05/Ch from diseased chickens. This NDV strain showed high genomic similarity to the genotype III ND vaccine strain Mukteswar but exhibited increased virulence following intravenous inoculation. The HN protein has been identified as the key virulence factor for JS/7/05/Ch [26]. Moreover, monocyte–macrophages have been demonstrated to be susceptible target cells of JS/7/05/Ch and Mukteswar [27]. However, the possible involvement of host molecules in viral infectivity remains unclear. In this study, we identified vimentin as an important host protein responsible for NDV infectivity in chicken macrophages. This study was the first to clarify the interaction between vimentin and NDV infection, offering crucial insights into the precise mechanism behind the diverse pathogenicity of genotype III NDVs.

## Materials and methods

### Viruses, cells, and antibodies

**Figure 1:**
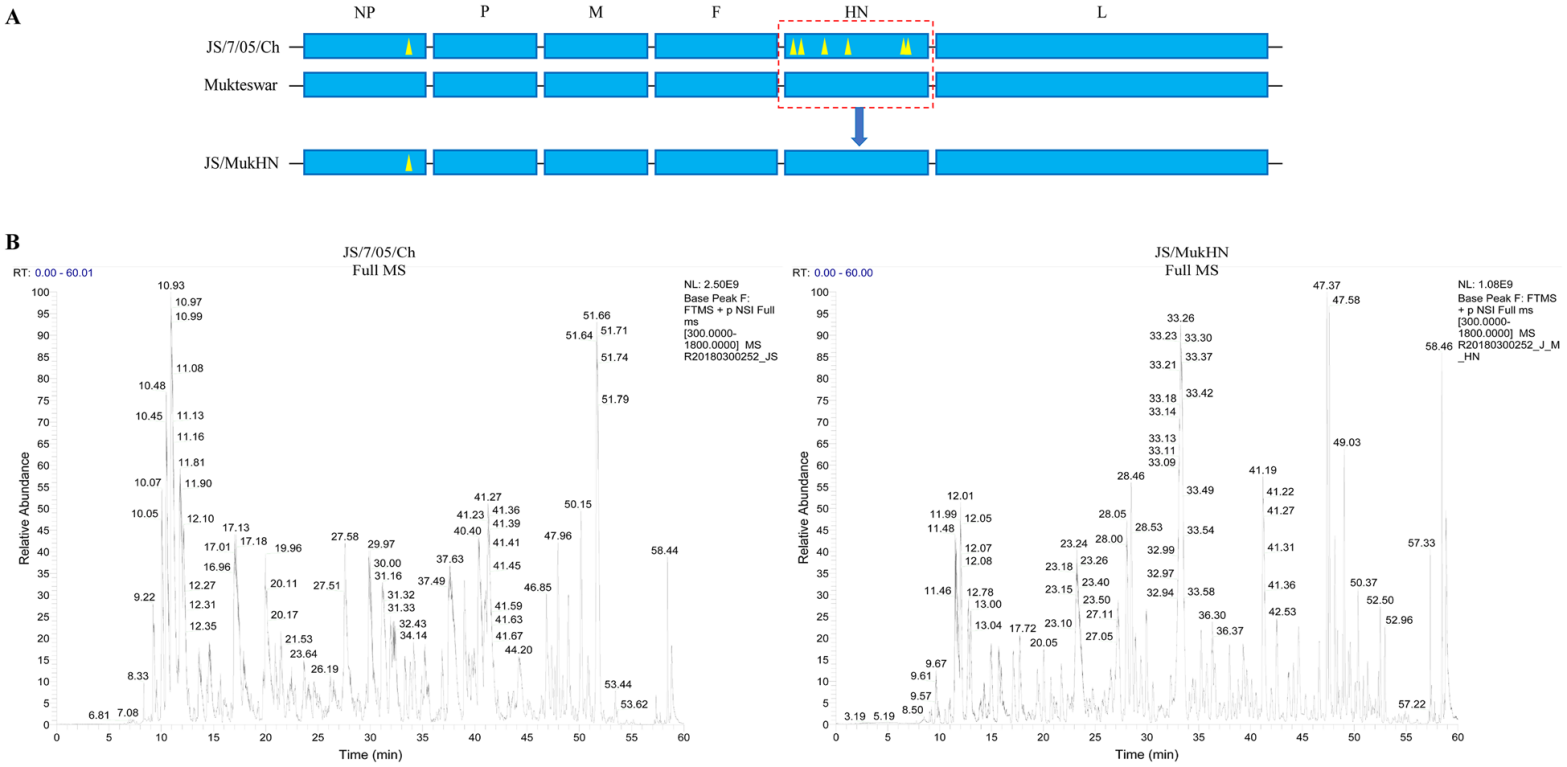
**Schematic representation of the model viruses and LC‒MS/MS spectra obtained in this study.** **A** Three NDV strains were utilized as model viruses in this study: the vaccine strain Mukteswar, the vaccine variant strain JS/7/05/Ch, and the HN replacement strain JS/MukHN, which is a derivative of JS/7/05/Ch carrying the HN gene from Mukteswar. The aa mutations in NDV NP and HN are marked with yellow triangles and include P438S in NP and N19S, S29T, M145T, V266I, A494D, and E495K in HN. **B** The LC‒MS/MS spectra were obtained for the immunoprecipitated protein samples. The protein samples obtained from the immunoprecipitates were prepared after the infection of JS/7/05/Ch and JS/MukHN. Subsequently, the samples underwent further processing and analysis using the LC‒MS/MS system. Coimmunoprecipitation assays were performed to investigate the interaction between NDV HN and vimentin.

The HN protein of the velogenic field isolate JS/7/05/Ch (GenBank: FJ430159.1), which evolved from the mesogenic ND vaccine strain Mukteswar (GenBank: JF950509.1), exhibited six amino acid (aa) variations. The six amino acid variations in HN are as follows: N19S, A29T, M145T, V266I, A494D, and E495K. Based on these two parental viruses, we previously generated a HN-replacement chimeric NDV JS/MukHN strain by a reverse genetics system (Figure 1A). With these NDVs as model viruses, NDV was propagated in the allantoic cavity of 10-day-old specific-pathogen-free (SPF) chicken embryos. The allantoic fluid was harvested 48 h after inoculation and stored at −70 °C for further characterization. Chicken macrophage (HD11) cells and chicken embryo fibroblasts (CEFs) were preserved and cultured in our laboratory. The half-maximal tissue culture infective dose (TCID_50_) of NDV was subsequently determined. To achieve inefficient viral replication, viral suspensions were irradiated with ultraviolet (UV) light as described previously [28]. Viral inactivation was then confirmed by validating the lack of replication in 9-day-old chicken embryonated eggs and in CEF monolayer cultures. The antibodies used in this study were as follows: mouse anti-NDV NP (provided by our laboratory), mouse anti-NDV HN (Santa Cruz Biotechnology, USA), rabbit anti-vimentin (Bioss, China), mouse anti-dsRNA (English & Scientific Consulting, Hungary), anti-mouse or anti-rabbit IgG (Beyotime Biotech, China), mouse anti-β-actin (TransGen Biotech, China), and anti-mouse or anti-rabbit IgG (secondary antibody; CWBIO, China).

### Immunoprecipitation and mass spectrometry analysis

A pair of viruses expressing different HN proteins, JS/7/05/Ch and JS/MukHN, were used to infect cells at a multiplicity of infection (MOI) of 1. Twenty-four hours post-infection (hpi), the cells were washed with PBS and lysed using 3 mL of ice-cold RIPA buffer (Beyotime Biotech, China) at 4 ℃ for 10 min. The cells were then disrupted by repeated aspiration through a needle and transferred into a 15 mL conical centrifuge tube. The cell culture plate was washed with an additional 1.0 mL of ice-cold RIPA buffer, which was then combined with the original lysate. Subsequently, these cell lysates were centrifuged at 10 000 × *g* for 10 min at 4 ℃. The supernatant was transferred into a fresh 15 mL conical centrifuge tube on ice. The cell lysate was precleaned by incubation with 1.0 µg of normal mouse IgG and 20 µL of resuspended Protein A/G PLUS-Agarose (Santa Cruz Biotechnology, USA) at 4 ℃ for 30 min. Afterwards, the lysate was centrifuged at 1000 × *g* for 5 min, and the supernatant was transferred into a fresh 15 mL conical centrifuge tube and incubated with an anti-NDV HN primary antibody for 1 h at 4 ℃. The extract was then incubated with 20 μL of resuspended Protein A/G PLUS-Agarose at 4 ℃ on a rotating device overnight. After centrifugation of the extract at 1000 × *g* for 5 min at 4 ℃, the supernatant was carefully aspirated and discarded. Finally, the acquired immunoprecipitated products were submitted to Shanghai Applied Protein Technology Co. Ltd for subsequent sample processing and identification by mass spectrometry. In brief, the polypeptide samples were digested with trypsin and analysed by LC‒MS/MS. Later, the LC‒MS/MS data were further evaluated using mass spectrometry matching software (MASCOT software) to obtain qualitative identification information for the target polypeptides.

### Western blot analysis

Cells were lysed in RIPA buffer supplemented with the proteinase inhibitor PMSF (Beyotime Biotech, China) at 24 hpi. The total protein concentration in the cell lysate was then measured using the BCA Protein Assay Kit (Beyotime Biotech, China). The denatured proteins were separated using 10% SDS‒PAGE and further transferred to polyvinylidene difluoride (PVDF) membranes. Subsequently, the PVDF membranes were blocked and incubated with diluted primary and secondary antibodies. Detection was performed by incubating the membranes with a chemiluminescent substrate and exposure in a dark room with a ChemiDoc Imager (Bio-Rad Laboratories, USA). Finally, the greyscale values of the bands were determined with ImageJ 1.48v software.

### Coimmunoprecipitation of NDV HN and vimentin

A coimmunoprecipitation (co-IP) assay was performed to identify the interaction of NDV HN and vimentin. Briefly, HD11 cells cultured in 6-well plates were transfected with the indicated plasmids for 24 h. These plasmids included eukaryotic expression plasmids for the prototypic (pcDNA3.1-MHN) or mutant (pcDNA3.1-JHN) HN protein and the empty pcDNA3.1, which were provided by our laboratory. Then, the cells were washed with ice-cold PBS, lysed with RIPA buffer, disrupted by repeated aspiration, and precleaned using normal mouse or rabbit IgG. The precleaned supernatant was incubated with an anti-NDV HN antibody, an anti-vimentin antibody, or normal IgG for 1 h at 4 °C. Thereafter, the extracts were incubated with resuspended Protein A/G PLUS-Agarose and further processed as described above. Later, the acquired coimmunoprecipitated products were washed with ice-cold PBS and resuspended in 40 μL of 1 × electrophoresis sample buffer. The samples were finally boiled for 5 min and analysed by Western blotting.

### RNA interference

The small interfering RNA (siRNA) targeting vimentin (sense 5′-3′: GGAUGUUUCUAAGCCUGAUT, antisense 5′-3′: AUCAGGCUUAGAAACAUCCTT) was synthesized by GenePharma (China) and named siRNA-VIM. A nontargeting siRNA (siRNA-NC) was used as a negative control. Cells cultured in 24-well plates were transfected with 60 pmol of siRNA using Polyfect Transfection Reagent (QIAGEN) according to the manufacturer’s protocol. The siRNA-transfected cells were identified by Western blotting at 24 h post-transfection (hpt).

### Overexpression of vimentin

A pair of primers (VIM-F: CCTCGAGGATGAGCTTCACCAGCAGCAAGAAC, VIM-R: GGGTACCCTTACTCCAAGTCATCGTGATGCTG) was designed for amplification of the full-length vimentin gene based on the published sequence of avian-derived vimentin (GenBank ID: NM_001048076). The vimentin gene was amplified from cDNA of HD11 cells with VIM-F and VIM-R. The amplified vimentin fragment was then successively inserted into the blunt-zero vector and pcDNA3.1 vector at two restriction sites, *Xho*I and *Eco*RI. The constructed vimentin expression plasmid, pcDNA3.1-VIM, was further confirmed by enzyme digestion and Sanger sequencing. Additionally, cells cultured in 12-well plates were transfected with 1 μg of the pcDNA3.1-VIM plasmid for 24 h, and the equivalent amount of empty pcDNA3.1 vector was used as a control. The transfected cells were further identified by Western blotting.

### Cell viability assay

Cell viability was examined following vimentin knockdown or overexpression. Cells cultured in 96-well plates were transfected with siRNA-VIM or pcDNA3.1-VIM for the indicated times. The infected cells were incubated with CCK-8 solution (CCK-8 assay kit, Beyotime Biotech, China). The absorbance was finally measured at 450 nm using a microplate reader after incubation for 1 h.

### Immunofluorescence assay (IFA)

HD11 cells were transfected with each siRNA or plasmid in 12-well cell culture plates. The transfected cells were sequentially washed with PBS, fixed with 4% paraformaldehyde, permeabilized with Triton X-100 (Beyotime Biotech, China), and blocked with 5% BSA. Subsequently, the cells were incubated with an anti-vimentin or anti-NP antibody at 4 ℃ overnight and with a FITC-conjugated (TransGen Biotech, China) or AF594-conjugated (Bioss, China) secondary antibody at 37 °C for 1 h. The cells were finally observed and photographed with a fluorescence microscope, with nuclei stained with DAPI (Beyotime Biotech, China).

### Confocal microscopy

Cells cultured on 14-mm coverslips were infected with NDVs following treatment with siRNAs, plasmids, or drugs for the indicated times. The cells were washed with PBS, fixed with 4% paraformaldehyde, permeabilized with Triton X-100 (Beyotime Biotech, China), and blocked with 5% BSA. The cells were then incubated with the primary antibody at 4 °C overnight and with a FITC-conjugated (TransGen Biotech, China) or AF594-conjugated (Bioss, China) secondary antibody at 37 °C for 1 h. Thereafter, nuclei were stained with DAPI (Beyotime Biotech, China), and the coverslips were mounted with PBS containing 50% glycerol. Finally, the cells were examined under a Leica LP8 confocal microscope.

### Drug treatment assay

Cells cultured on 14-mm coverslips were infected with NDV at an MOI of 1 at 37 ℃. After adsorption for 1 h, the cells were washed with PBS and cultured in DMEM containing 1.5% 3,3’-iminodipropionitrile (IDPN) (Sigma‒Aldrich, USA). Thereafter, the cells were processed and examined under a Leica LP8 confocal microscope as described above.

### Viral replication assay

NDV replication was evaluated following vimentin knockdown or overexpression in cells. Briefly, cells cultured in 24-well plates were transfected with siRNA-VIM or pcDNA3.1-VIM as described above. At 24 hpt, the cells were infected with each of the three genotype III NDVs at an MOI of 1 for the indicated time. The expression level of the NDV NP protein was measured by Western blotting and IFA at 24 hpi. The viral titres in the cell supernatants were determined by a TCID_50_ assay at 6, 12, and 24 hpi.

### Viral attachment characterization

To investigate the impact of vimentin on viral attachment, HD11 cells were exposed to NDVs after pretreatment with an anti-vimentin antibody. First, to validate the efficacy of the anti-vimentin antibody in blocking cell surface vimentin, the HN-vimentin interaction level was assessed after anti-vimentin antibody treatment. Briefly, HD11 cells in 6-well cell culture plates were pretreated with an anti-vimentin antibody and then exposed to JS/7/05/Ch, JS/MukHN, and Mukteswar at an MOI of 1. The infected cells were harvested and lysed at 24 hpi, and the assay was conducted according to the manufacturer’s instructions for the Co-Immunoprecipitation Kit (Santa Cruz Biotechnology, USA). The immunoprecipitated proteins were then identified and analysed by Western blotting using an anti-HN antibody, an anti-vimentin antibody or normal mouse IgG.

After verifying the blocking efficiency of the anti-vimentin antibody, HD11 cells were cultured in 6-well plates and infected with NDV strains (MOI = 1) at 4 ℃ for 1 h after pretreatment with either an anti-vimentin antibody or normal rabbit IgG. Subsequently, the cells were washed using ice-cold PBS solution (pH = 7.2) containing 2% FBS. The infected cells were then incubated with an anti-NDV HN primary antibody at 4 ℃ overnight prior to incubation with a FITC-conjugated secondary antibody (Beyotime Biotech, China) at 37 ℃ for 1 h. Finally, the virus-bound cells were digested using pancreatic enzymes and analysed using flow cytometry (CytoFLEX, Beckman, China).

### Viral internalization characterization

An acidic PBS solution (pH = 1.5) can effectively elute virus particles from the cell surface that have not yet been internalized into cells. To assess the role of vimentin in viral internalization, HD11 cells were exposed to NDVs following vimentin knockdown. Briefly, HD11 cells were cultured in 6-well plates and transfected with siRNA-VIM or siRNA-NC for 24 h. The transfected cells were subsequently infected with NDV (MOI = 1) at both 4 ℃ and 37 ℃ for 1 h. Following infection, the cells were washed with ice-cold acidic PBS (pH = 1.5) to remove the attached but uninternalized virions. Next, the cells were incubated with an anti-NDV NP primary antibody overnight at 4 ℃ prior to a 1-h incubation at 37 ℃ with a FITC-conjugated secondary antibody (Beyotime Biotech, China). Finally, the cells with internalized virus were detected using flow cytometry (CytoFLEX, Beckman, China).

### Cell fusion assay

The fusion of the NDV strains were examined as described previously [29]. Briefly, HD11 cells were infected with NDV strains at an MOI of 1 for 24 h after transfection with siRNA. The infected cells were then fixed with 4% paraformaldehyde after three washes with ice-cold PBS. Syncytium formation was observed after staining with Giemsa solution (Beyotime Biotech, China). The fusion index was calculated as the ratio of the number of nuclei in the syncytia to the total number of nuclei in the field. The fusion index values of the viruses were normalized to the value for the siRNA-NC-Mukteswar group, which was considered to be 1.

### Viral release characterization

The release of NDVs was analysed by measuring the HA titres in the supernatant and cells. Briefly, HD11 cells cultured in 24-well plates were infected with NDV strains at an MOI of 1 after transfection with siRNA-VIM or siRNA-NC. The infected cells were maintained in culture in 0.5 mL DMEM supplemented with 2% serum. Afterwards, the cells were digested using pancreatin after the collection of supernatants at the indicated time, and the digested cells were resuspended in 0.5 mL of DMEM supplemented with 2% serum. Finally, viral release was quantified by calculating the ratio of the HA titre in the supernatant to the sum of the HA titres in the supernatant and cells.

### Statistical analysis

One-way or two-way analysis of variance (ANOVA) was used to determine statistical significance. Statistical analyses were conducted with GraphPad Prism 7.00 software. *p* < 0.05 was considered to indicate a statistically significant difference.

## Results

### Cellular vimentin differentially interacts with NDV HN proteins

NDV HN has been widely reported to be involved in viral infection; however, the interactions between HN and host proteins remain unknown. To investigate the host proteins that interact differentially with NDV HN, we selected a pair of genotype III NDVs with distinct HN proteins, JS/7/05/Ch and JS/MukHN, for mass spectrometry analysis (Figure 1B). Total protein in the NDV-infected cell lysate subjected to coimmunoprecipitation with an anti-NDV HN antibody. The immunoprecipitated proteins were identified by LC‒MS/MS, which revealed differential interactions between several host proteins and the prototypic HN protein (Mukteswar-type HN) versus the mutant HN protein (JS/7/05/Ch-type HN) (for the raw mass spectrometry data, see Additional files 1 and 2). Among these host proteins, vimentin was identified with high confidence, with 3 peptides unique to the protein and 9.44% sequence coverage. As demonstrated by the results of LC‒MS/MS analysis, the prototypic HN protein displayed a robust interaction with vimentin, whereas the mutant HN protein exhibited no interaction with vimentin (Table 1).

**Table 1:** **Screening of differentially interacting proteins by LC‒MS/MS**

Accession	Protein name	Host	Σ#Coverage	Σ# Unique Peptides	Score JS/7/05/Ch	Score JS/MukHN
A0A1L1RXL9	Vimentin	*Gallus gallus*	9.44	3	–	150.09
A0A1D5P5D1	Actin	*Gallus gallus*	6.39	2	–	82.96
F1NDN6	Keratin 12	*Gallus gallus*	5.11	1	73.57	126.51
A0A1L1RN85	Tubulin beta chain	*Gallus gallus*	4.81	1	–	51.01
Q6PVZ3	alpha-keratin IIC	*Gallus gallus*	4.41	1	–	105.03
E1BZ05	Desmin	*Gallus gallus*	4.31	1	–	119.64
E1BVU7	Sperm antigen with calponin homology and coiled-coil domains 1	*Gallus gallus*	0.65	1	–	37.79
F1NV02	Apolipoprotein B	*Gallus gallus*	0.26	1	19.55	–

**Figure 2:**
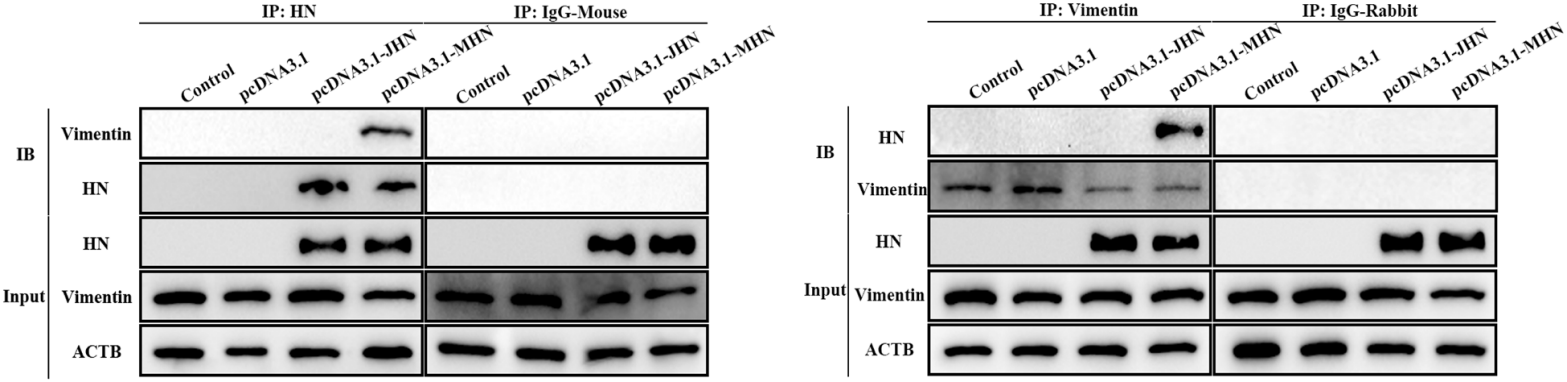
**Interactions between different HN proteins and vimentin.** HD11 cells in 6-well cell culture plates were transfected with HN-expressing (pcDNA3.1-JHN or pcDNA3.1-MHN) or empty (pcDNA3.1) plasmid. Transfected cells were harvested and lysed 24 hpt, and the experiment was conducted according to the manufacturer’s instructions for the Co-Immunoprecipitation Kit (Santa Cruz Biotechnology, USA). The immunoprecipitated proteins were then identified and analysed by Western blotting using an anti-HN antibody, an anti-vimentin antibody or normal mouse/rabbit IgG. Three groups of cells were included in the co-IP experiment: the input group, antibody group and IgG group. pcDNA3.1-JHN: pcDNA3.1 plasmid containing the sequence encoding the JS/7/05/Ch-type HN protein. pcDNA3.1-MHN: pcDNA3.1 plasmid containing the sequence encoding the Mukteswar-type HN protein.

To verify the specific interactions between vimentin and HN proteins, coimmunoprecipitation assays were performed on chicken macrophages, which have been reported to be susceptible target cells of genotype III NDVs. HD11 chicken macrophages were transfected with the indicated plasmids expressing HN proteins and subsequently subjected to co-IP assays. As shown in Figure 2, cellular vimentin was coimmunoprecipitated with the prototypic HN protein but not with the mutant HN protein or in the empty vector control group. This result indicated that vimentin interacted with the prototypic HN protein but not with the mutant HN protein. As expected, cellular vimentin could not be coimmunoprecipitated with HN proteins in the normal mouse and rabbit IgG control groups. The input groups showed efficient HN expression in cells transfected with pcDNA3.1-JHN and pcDNA3.1-MHN. These findings from the co-IP assay further confirmed the mass spectrometry results. Taken together, these results suggest that the prototypic and mutant HN proteins of genotype III NDVs differentially interact with cellular vimentin.

### NDVs expressing different HN proteins differentially induce vimentin rearrangement

**Figure 3:**
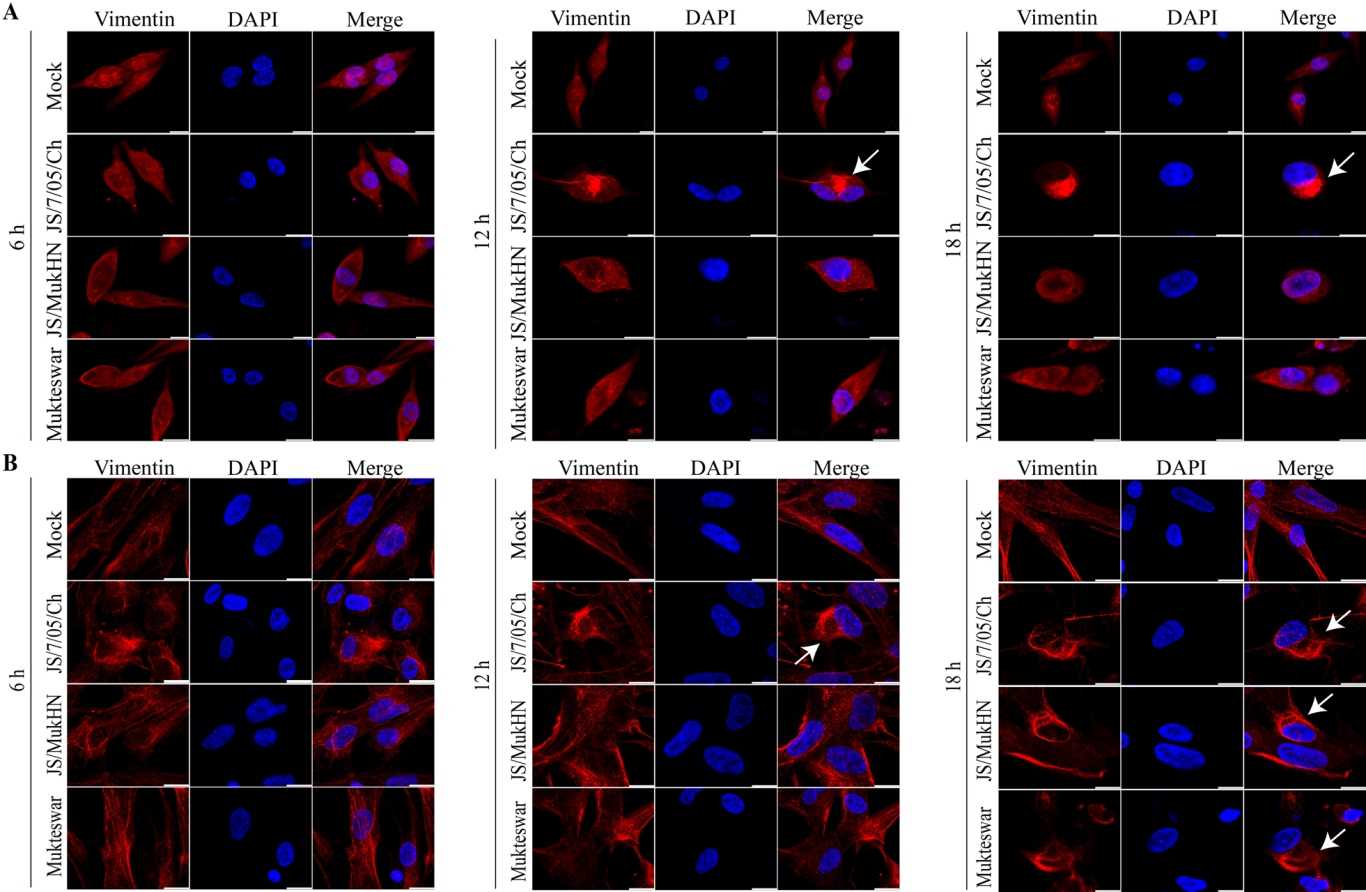
**The regulation of vimentin rearrangement by NDV infection.** NDV infection induces differential vimentin rearrangement in HD11 cells (**A**) and CEFs (**B**). Cells were infected with JS/7/05/Ch, JS/MukHN, or Mukteswar at an MOI of 1 for 6, 12, and 18 h. The infected cells were fixed with cold 4% paraformaldehyde, permeabilized with Triton X-100, and blocked with 5% BSA. Vimentin (red) was subsequently visualized using an anti-vimentin antibody on a confocal microscope, with the nuclei stained with DAPI (blue). The mock-infected cells were assayed in parallel as a control. Images were captured at different magnifications to obtain more precise fields of view. The white arrowheads indicate rearranged vimentin. Scale bars, 10 μm.

Viral infection can trigger the rearrangement of vimentin filaments, an event closely related to virus replication [30, 31]. To investigate the impact of NDV infection on the structure of vimentin filaments, we conducted observations of the vimentin filament structure after NDV infection. In mock-infected cells, vimentin filaments exhibited a widely distributed long network structure throughout the cytoplasm. In JS/7/05/Ch-infected HD11 cells, the vimentin filaments were dense and short and extensively accumulated around the nucleus from 12 hpi. In contrast, there was no apparent rearrangement of vimentin in either JS/MukHN- or Mukteswar-infected HD11 cells (Figure 3A). In CEFs, however, all the NDVs could induce rearrangement of vimentin after 18 hpi, and no significant difference was observed (Figure 3B). Thus, these results demonstrate that the velogenic variant NDV strain exhibits a significant advantage in inducing vimentin rearrangement in HD11 cells.

### NDV replication and vimentin rearrangement are reciprocally dependent

**Figure 4:**
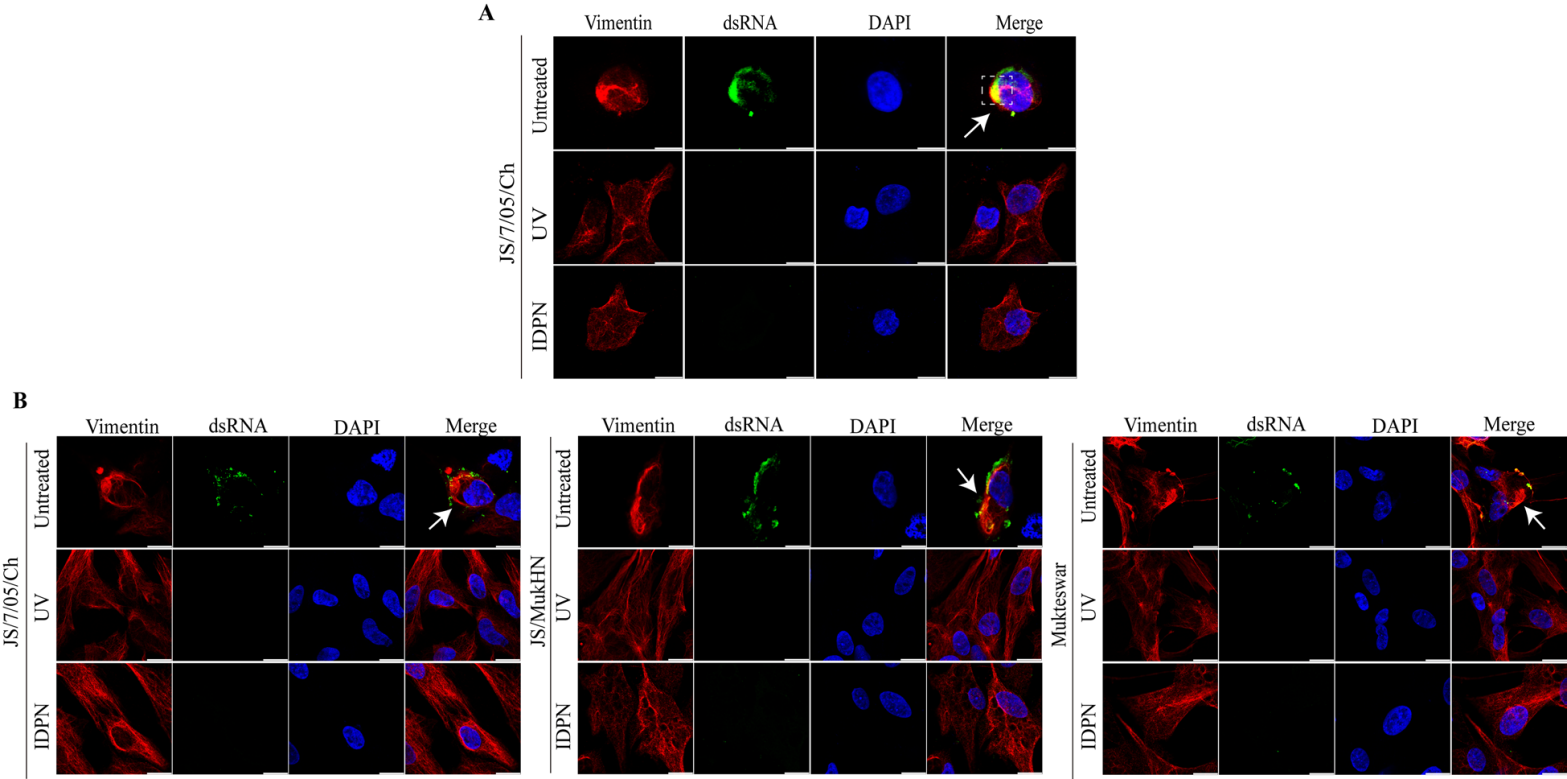
**Reciprocal dependency between NDV infection and vimentin rearrangement.** NDV infection induces differential vimentin rearrangement in HD11 cells (**A**) and CEFs (**B**). Cells were infected with non-UV-inactivated NDV or UV-inactivated NDV at an MOI of 1 for 18 h and were also pretreated with IDPN prior to being infected with NDV. The infected cells were then fixed with cold 4% paraformaldehyde, permeabilized with Triton X-100, and blocked with 5% BSA. NDV dsRNA (green) and vimentin (red) were visualized using specific antibodies on a confocal microscope, with the nuclei stained with DAPI (blue). Images were captured at 630 × magnification. The white arrowheads indicate rearranged vimentin. The white dashed box indicates colocalization of rearranged vimentin filaments and viral dsRNA. Scale bars, 10 μm.

Successful virus replication is essential for the rearrangement of vimentin, which in turn can regulate virus replication [32]. To clarify the relationship between NDV replication and vimentin rearrangement, we employed IDPN treatment to disrupt vimentin rearrangement and UV treatment to deactivate the virus. Both viral replication and vimentin rearrangement were subsequently monitored. A previous study demonstrated that vimentin rearrangement was observed exclusively in JS/7/05/Ch-infected HD11 cells, whereas no such rearrangement was observed in Mukteswar- and JS/MukHN-infected HD11 cells. Therefore, we next investigated the relationship between JS/7/05/Ch replication and vimentin rearrangement in HD11 cells. In untreated HD11 cells, JS/7/05/Ch effectively induced vimentin rearrangement and widespread production of viral dsRNA in the cytoplasm. Importantly, there was noticeable colocalization between the rearranged vimentin filaments and viral dsRNA after infection with JS/7/05/Ch in the cytoplasm. In contrast, JS/7/05/Ch was unable to trigger vimentin rearrangement and dsRNA production in HD11 cells treated with IDPN. Thus, these findings suggest that the rearrangement of vimentin is essential for the generation of viral dsRNA in HD11 cells, which serve as a replication factory for NDV. Additionally, UV-inactivated JS/7/05/Ch did not induce vimentin rearrangement or dsRNA production in HD11 cells, further highlighting the importance of virus replication in the process of vimentin rearrangement (Figure 4A). These findings were also validated in CEFs infected with NDV, where vimentin rearrangement and virus replication were found to be mutually dependent. Briefly, we assessed the relationship between the replication of three genotype III NDVs and vimentin rearrangement in CEFs. We found that all three NDVs effectively caused significant vimentin rearrangement and resulted in dsRNA production in untreated CEFs. In contrast, all three NDVs failed to induce evident vimentin rearrangement and dsRNA production in IDPN-treated CEFs. Additionally, UV-inactivated NDVs did not induce vimentin rearrangement or dsRNA production in CEFs. It is worth noting that in CEFs, there was no apparent colocalization between rearranged vimentin and viral dsRNA, unlike in HD11 cells. This result further emphasizes that genotype III NDVs primarily target chicken macrophages as susceptible host cells (Figure 4B). In summary, these results suggest a mutually dependent relationship between NDV replication and vimentin rearrangement.

### Vimentin mediates differential effects on the replication of NDVs

**Figure 5:**
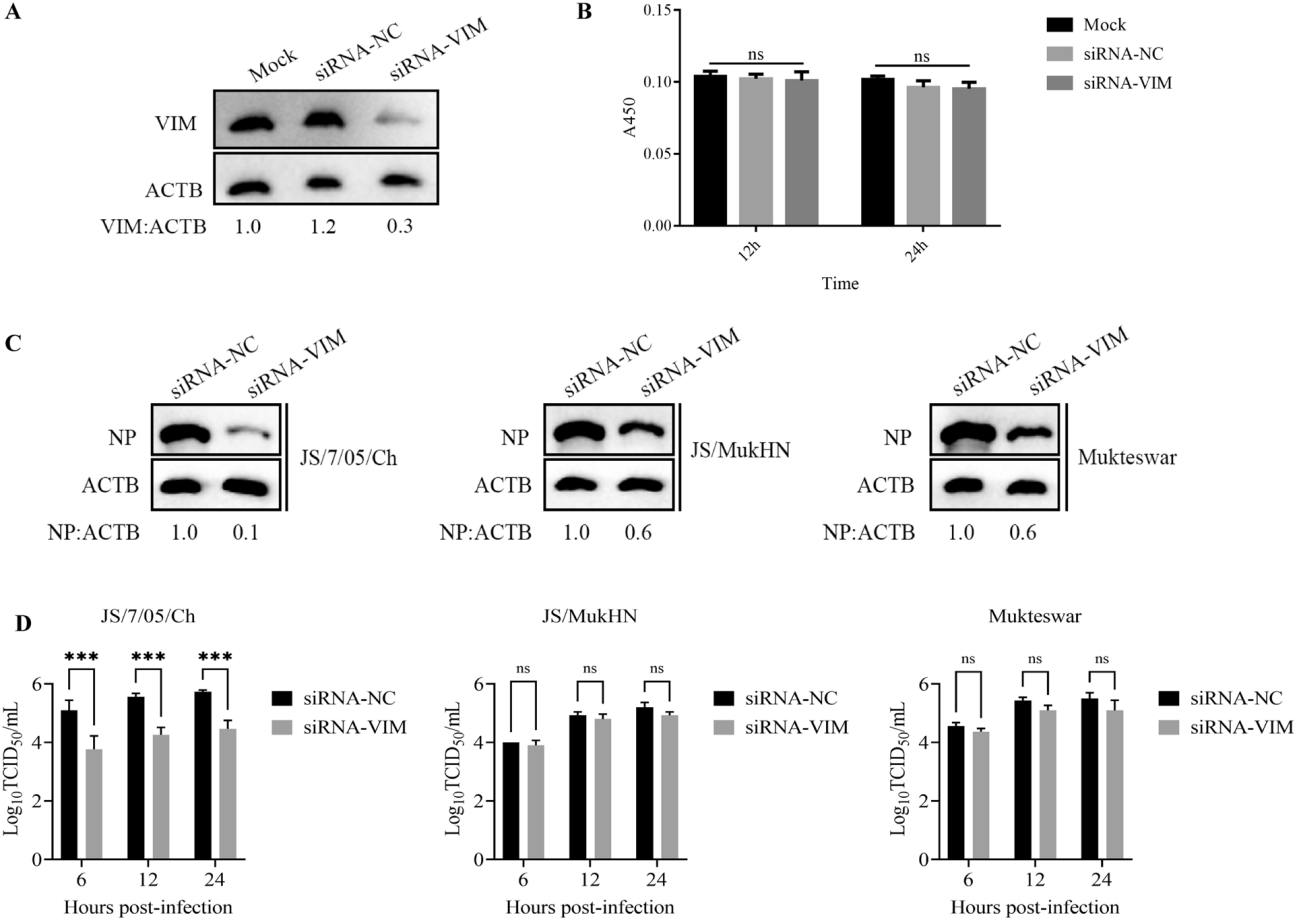
**Effects of vimentin knockdown on NDV replication.** **A** HD11 cells were transfected with siRNA targeting vimentin (siRNA-VIM) or control siRNA (siRNA-NC) for 24 h. The knockdown efficiency of vimentin was determined by Western blotting using an anti-vimentin antibody. Vimentin protein levels were normalized to ACTB protein levels and are shown relative to the levels in the Mock group. The band greyscale values were determined by ImageJ 1.4 software. **B** HD11 cells were transfected with siRNA-VIM or siRNA-NC for 12 and 24 h. The transfected cells were harvested and used for a CCK-8 assay according to the manufacturer's instructions. The mock-transfected cells were assayed in parallel as a control. **C** HD11 cells were transfected with siRNA-VIM or siRNA-NC for 24 h and then infected with JS/7/05/Ch, JS/MukHN, or Mukteswar at an MOI of 1 for 24 h. The infected cells were harvested and lysed using RIPA buffer supplemented with the proteinase inhibitor PMSF. NDV NP expression levels were then measured by Western blotting with specific antibodies. The band greyscale values were determined by ImageJ 1.4 software. NP protein levels were normalized to ACTB protein levels and are shown relative to those in siRNA-NC-transfected cells. **D** HD11 cells were transfected with siRNA-VIM or siRNA-NC for 24 h and then infected with JS/7/05/Ch, JS/MukHN, or Mukteswar at an MOI of 1 for 6, 12, and 24 h. The supernatant of the infected cells was used for the determination of viral titres by a TCID50 assay. The statistical graphs were generated using GraphPad Prism 9.0 software. ***p < 0.001; ns indicates no significance.

To further explore the role of vimentin in NDV replication, we quantified virus replication following vimentin knockdown or overexpression. For the vimentin knockdown assay, HD11 cells were transfected with siRNA-VIM or siRNA-NC for 24 h. The knockdown efficiency of siRNA-VIM was verified by Western blotting. Vimentin expression was significantly reduced in cells transfected with siRNA-VIM compared to those transfected with siRNA-NC (Figure 5A). Moreover, cell viability remained unchanged upon siRNA transfection (Figure 5B). These results indicated the successful knockdown of vimentin in HD11 cells. The transfected cells were then infected with the three NDV strains at an MOI of 1 for 24 h. Virus replication was quantified by Western blotting and viral titration assays. As shown in Figure 5C, the expression of JS/7/05/Ch NP showed a 90% reduction in vimentin-knockdown cells compared to negative control cells. In contrast, the expression of Mukteswar NP and JS/MukHN NP exhibited a decrease of only 40% following vimentin knockdown. To determine the differences in replication time between the virus strains, we further measured the viral titre in the supernatant at multiple time points following vimentin knockdown. As shown in Figure 5D, vimentin knockdown led to a notable reduction in the viral titre specifically in the supernatant of JS/7/05/Ch-infected cells from 6 to 24 hpi (*p* < 0.001), whereas no significant decrease in the viral titre was observed in the supernatants of other infected cells during infection (*P* > 0.05).

**Figure 6:**
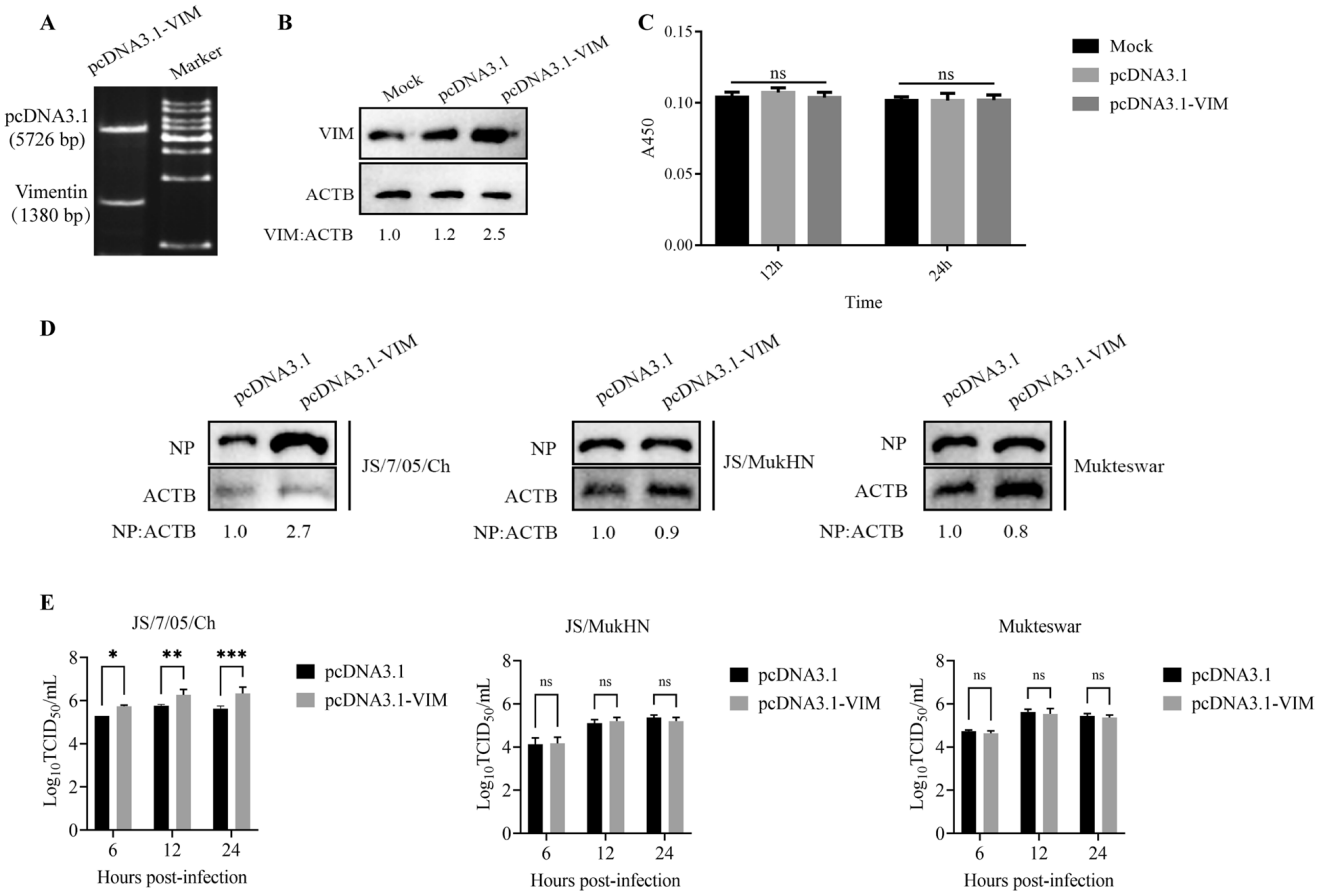
**Effects of vimentin overexpression on NDV replication.** **A** Confirmation of pcDNA3.1-VIM by restriction analysis. Vimentin fragment: 1380 bp; empty pcDNA3.1 vector: 5726 bp; marker: 1 kb DNA Ladder. **B** HD11 cells were transfected with a plasmid containing the full-length vimentin gene sequence (pcDNA3.1-VIM) or the empty plasmid (pcDNA3.1) for 24 h. The overexpression efficiency of vimentin was assessed by Western blotting using an anti-vimentin antibody. The band greyscale values were determined by ImageJ 1.4 software. Vimentin protein levels were normalized to ACTB protein levels and are shown relative to the levels in the Mock group. **C** HD11 cells were transfected with pcDNA3.1-VIM or pcDNA3.1 for 12 and 24 h. The transfected cells were harvested and used for a CCK-8 assay according to the manufacturer's instructions. The mock-transfected cells were assayed in parallel as a control. **D** HD11 cells were transfected with pcDNA3.1-VIM or pcDNA3.1 for 24 h and then infected with JS/7/05/Ch, JS/MukHN, or Mukteswar at an MOI of 1 for 24 h. The infected cells were harvested and lysed using RIPA buffer supplemented with the proteinase inhibitor PMSF. NDV NP expression levels were then measured by Western blotting with specific antibodies. The band greyscale values were determined by ImageJ 1.4 software. NP protein levels were normalized to ACTB protein levels and shown relative to pcDNA3.1. **E** HD11 cells were transfected with pcDNA3.1-VIM or pcDNA3.1 for 24 h and then infected with JS/7/05/Ch, JS/MukHN, or Mukteswar at an MOI of 1 for 6, 12, and 24 h. The supernatant of the infected cells was used for the determination of viral titres by a TCID50 assay. The statistical graphs were generated using GraphPad Prism 9.0 software. *p < 0.05; **p < 0.01; ***p < 0.001; ns indicates no significance.

For the vimentin overexpression assay, we successfully confirmed the pcDNA3.1-VIM plasmid through endonuclease digestion and agarose electrophoresis (vimentin fragment: 1380 bp; empty pcDNA3.1 vector: 5726 bp) (Figure 6A). Subsequently, HD11 cells were transfected with either pcDNA3.1-VIM or empty pcDNA3.1 vector for 24 h, and the transfection efficiency of pcDNA3.1-VIM was evaluated by Western blotting. The level of vimentin expression in the vimentin-overexpressing cells was significantly higher than that in the negative control cells (Figure 6B). In addition, cell viability remained unchanged after plasmid transfection (Figure 6C). These results indicated the successful overexpression of vimentin. Afterwards, cells overexpressing vimentin were infected with the three NDV strains at an MOI of 1 for 24 h, and virus replication was quantified as described above. As shown in Figure 6D, vimentin overexpression resulted in a 170% increase in JS/7/05/Ch NP expression in HD11 cells compared to negative control cells, while no increase in the expression of Mukteswar NP or JS/MukHN NP was observed. To determine the differences in replication time between the virus strains, we further measured the viral titre in the supernatant at multiple time points after vimentin overexpression. As shown in Figure 6E, vimentin overexpression led to a noteworthy increase in the viral titre in the supernatant of only JS/7/05/Ch-infected cells at 6 (*p* < 0.05), 12 (*p* < 0.01), and 24 (*p* < 0.001) hpi and not in that of the other infected cells (*p* > 0.05).

IFA was also conducted to assess the level of virus replication following vimentin knockdown or overexpression. The results of immunofluorescence analysis were consistent with those described above. In comparison to control cells, cells transfected with siRNA-VIM displayed reduced fluorescence, and those transfected with pcDNA3.1-VIM exhibited increased fluorescence. These results confirm the successful knockdown and overexpression of vimentin. Specifically, knockdown of vimentin resulted in a significant decrease in the fluorescence intensity of the NP protein, while overexpression of vimentin significantly increased the fluorescence intensity of the NP protein in JS/7/05/Ch-infected cells. However, no discernible alteration was noted in JS/MukHN- or Mukteswar-infected cells after vimentin knockdown or overexpression (Additional file 3). Hence, these findings suggest that vimentin exerts a substantial effect on the replication of the velogenic variant NDV strain, whereas it does not affect the mesogenic strain during infection.

### Vimentin does not significantly impact NDV attachment in HD11 cells

Considering that vimentin exhibited varying effects on the replication of NDVs, we hypothesized that vimentin could play an important role in NDV infection. To test this hypothesis, we conducted further investigation into the effects of vimentin during different stages of infection in HD11 cells. Initially, our focus was on the critical role of viral attachment as the first step in determining the success of infection [33]. To investigate the impact of vimentin on NDV attachment to the cell surface, we assessed the level of viral attachment through flow cytometry following treatment with an anti-vimentin antibody. First, the interaction between HN and vimentin was evaluated to validate the effectiveness of the anti-vimentin antibody in blocking cell surface vimentin. Here, we employed NDVs carrying the prototypic HN protein, known to interact with vimentin, for the co-IP assay. The results of the co-IP assay revealed that the prototypic HN protein exhibited a robust interaction with vimentin in cells treated with normal mouse IgG (Vimentin/HN: 1.9 and 1.7). Conversely, a less pronounced interaction between HN and vimentin was noted in cells treated with the anti-vimentin antibody (Vimentin/HN: 1.2 and 1.0) (Additional file 4). As anticipated, cellular vimentin did not coimmunoprecipitate with HN proteins in the normal mouse IgG control group. The input groups exhibited successful viral infection in cells that were exposed to JS/MukHN and Mukteswar. These findings indicate the successful blockade of cell surface vimentin by the anti-vimentin antibody.

**Figure 7:**
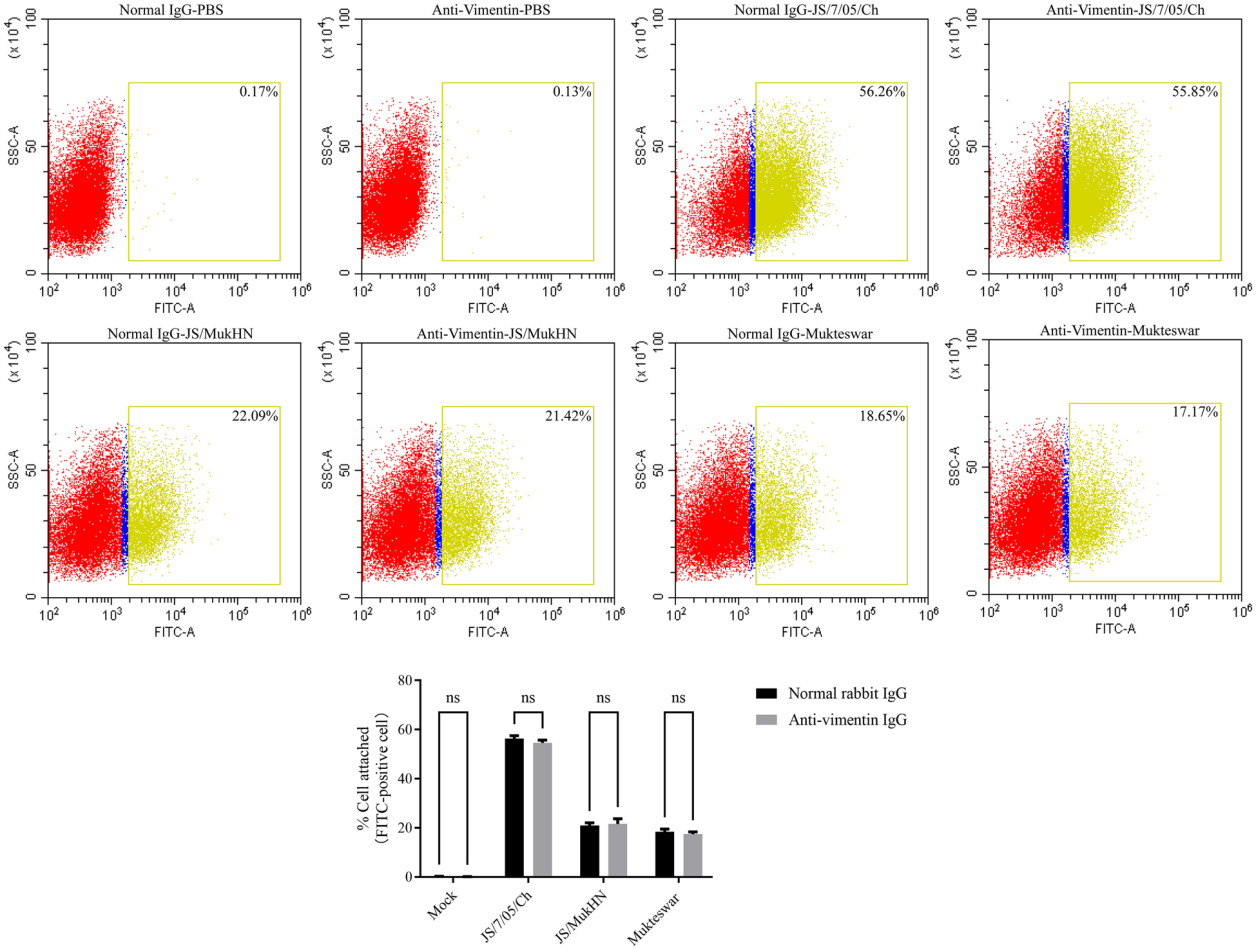
**Effects of vimentin on the attachment of NDV.** HD11 cells in 6-well cell culture plates were pretreated with an anti-vimentin antibody or normal rabbit IgG and then infected with NDV (MOI = 1) or treated with PBS at 4 ℃ for 1 h. The infected cells were washed with ice-cold PBS solution (pH = 7.2) containing 2% FBS. Then, the cells were incubated with a mouse anti-NDV HN primary antibody at 4 ℃ overnight and a FITC-conjugated secondary antibody at 37 ℃ for 1 h. The mock-infected cells were assayed in parallel as a control. The cells with NDV attachment were finally detected by flow cytometry, and flow cytometry scatter plots were generated using CytExpert 2.3 software. Positive cell populations were established based on granularity (SSC-A) and the FITC-A fluorescence channel. The ratio of the number of positive cells to the total number of cells was calculated and is displayed within the box. The statistical graphs were generated using GraphPad Prism 9.0 software. ns indicates no significance.

Here, FITC-positive cells were identified as cells with successful attachment of NDV. In the mock-infected groups, scarcely any cells showed positive FITC staining after treatment with normal IgG (0.17%) or anti-vimentin IgG (0.13%), indicating the absence of virus attachment in these groups. The NDV-infected groups exhibited comparable percentages of FITC-positive cells when treated with normal rabbit IgG and the anti-vimentin antibody. The percentages were as follows: JS/7/05/Ch: 56.26% versus 55.85%; JS/MukHN: 22.09% versus 21.42%; and Mukteswar: 18.65% versus 17.17%. Notably, there was no significant difference in the number of FITC-positive cells between the groups treated with normal rabbit IgG and the groups treated with the anti-vimentin antibody (*P* > 0.05) (Figure 7). This indicates that vimentin does not exert a significant effect on NDV attachment. Therefore, these results demonstrate that cell surface vimentin is not necessary for NDV attachment.

### Vimentin differentially regulates the processes of NDV internalization, fusion, and release in HD11 cells

**Figure 8:**
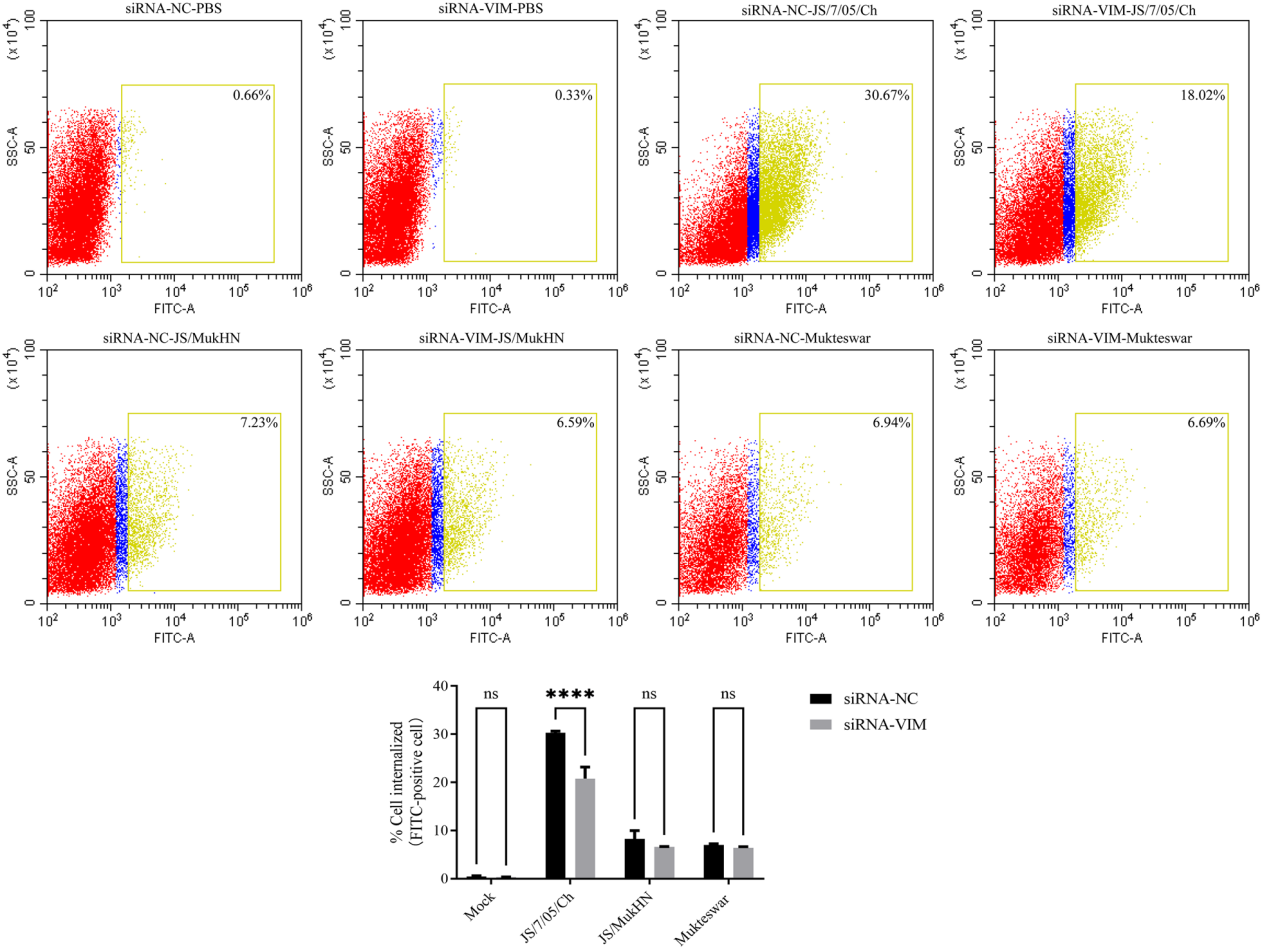
**Effects of vimentin on the internalization of NDV.** HD11 cells in 6-well cell culture plates were pretreated with siRNA-VIM or siRNA-NC. The pretreated cells were infected with 1 MOI JS/7/05/Ch, JS/MukHN or Mukteswar at 4 ℃ and 37 ℃ for 1 h, successively. The mock-infected cells were assayed in parallel as a control. The infected cells were washed with ice-cold acidic PBS (pH = 1.5) to remove the attached but not yet internalized virions. The cells were then incubated with a mouse anti-NDV NP primary antibody and secondary antibody conjugated to FITC. The NDV-internalized cells were finally detected by flow cytometry, and flow cytometry scatter plots were generated using CytExpert 2.3 software. Positive cell populations were established based on granularity (SSC-A) and the FITC-A fluorescence channel. The ratio of the number of positive cells to the total number of cells was calculated and is displayed within the box. The statistical graphs were generated using GraphPad Prism 9.0 software. ****p < 0.0001; ns indicates no significance.

To clarify the role of vimentin in the subsequent processes in NDV infection following viral attachment, we further investigated the effects of vimentin on NDV internalization, fusion, and release in HD11 cells. Viral internalization was analysed by flow cytometry. Here, FITC-positive cells were identified as cells with successful internalization by NDV. As shown in Figure 8, almost none of the cells in the mock-infected groups were FITC-positive following treatment with siRNA-VIM or siRNA-NC. In the NDV-infected groups, there was a significant reduction in FITC-positive cells in the JS/7/05/Ch-infected group following vimentin knockdown (*p* < 0.0001), whereas the proportion of FITC-positive cells in both the JS/MukHN- and Mukteswar-infected groups showed no significant difference following vimentin knockdown (*p* > 0.05). These findings indicate the significant impact of vimentin on the internalization process of the velogenic variant NDV strain.

**Figure 9:**
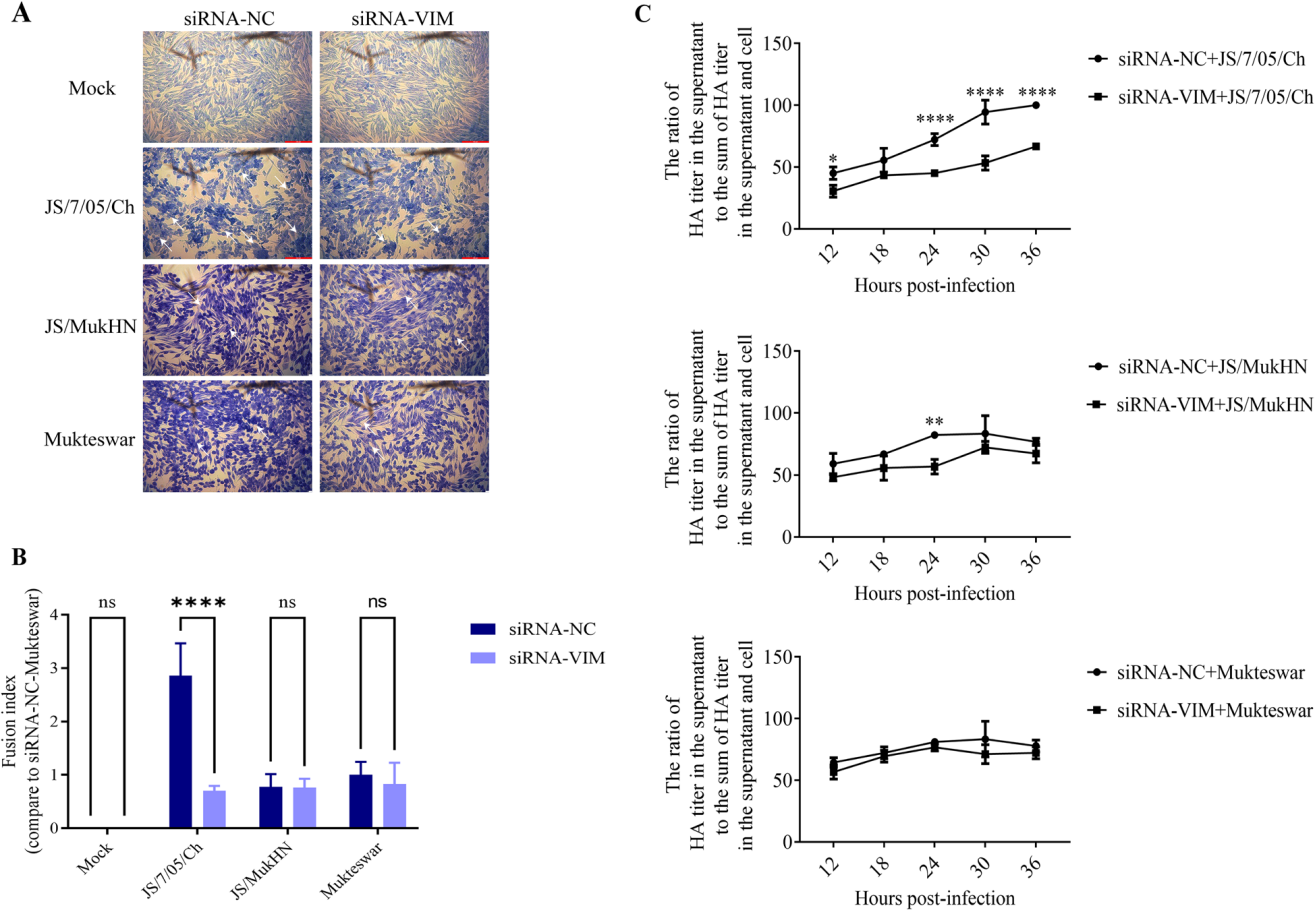
**Effects of vimentin on the fusion and release of NDV.** **A** HD11 cells in 6-well cell culture plates were transfected with siRNA-VIM or siRNA-NC for 24 h. The transfected cells were then infected with JS/7/05/Ch, JS/MukHN, or Mukteswar at an MOI of 1 for 24 h. After being washed three times with ice-cold PBS, the infected cells were subsequently fixed using 4% paraformaldehyde prior to observation of syncytia formation post-staining with Giemsa solution. The mock-infected cells were assayed in parallel as a control. Images were acquired under a microscope at 100 × magnification. Scale bars, 100 μm. The white arrowheads indicate syncytia. **B** The fusion index was calculated as the ratio of the number of nuclei within syncytia to the total number of nuclei in the visual field. The fusion index value for each experimental group was then normalized to the value in the siRNA-NC-Mukteswar group, which was established as the baseline value of 1. **C** HD11 cells in 24-well cell culture plates were transfected with siRNA-VIM and siRNA-NC for 24 h. The transfected cells were then infected with JS/7/05/Ch, JS/MukHN, or Mukteswar at an MOI of 1 for 12, 18, 24, 30, and 36 h. Viral release was quantified by calculating the ratio of the HA titre in the supernatant to the sum of the HA titres in the supernatant and cells. The statistical graphs were generated using GraphPad Prism 9.0 software. **p < 0.01; ****p < 0.0001; ns indicates no significance.

The effects of vimentin on the membrane fusion activity of NDV were assessed through the measurement of fusion indices. As shown in Figures 9A, B, the fusion activity of JS/7/05/Ch exhibited a significant reduction in vimentin-knockdown cells compared to control cells (*p* < 0.0001). However, the fusion activities of Mukteswar and JS/MukHN showed no significant changes following vimentin knockdown (*p* > 0.05). These results indicate the crucial role of vimentin in the fusion process of the velogenic variant NDV strain.

The impact of vimentin on the release of NDV was evaluated by HA titration. As shown in Figure 9C, the release of JS/7/05/Ch showed a significant decrease in vimentin-knockdown HD11 cells, particularly after 24 hpi (*p* < 0.0001), compared to that in control cells. In comparison, the release of JS/MukHN and Mukteswar varied little after vimentin knockdown and showed a significant decrease (*p* < 0.01) only in the JS/MukHN group at 24 hpi. These results indicated the important role of vimentin in the release process of the velogenic variant NDV strain. Taken together, these findings indicate that vimentin is responsible for mediating various steps in the infection process of the velogenic variant NDV strain but not the mesogenic strain.

## Discussion

The HN protein, an important envelope glycoprotein, is important for NDV infectivity and pathogenicity [34]. The mesogenic ND vaccine strain Mukteswar was initially isolated in India and subsequently attenuated through multiple passages in chicken embryos. During poultry vaccination, Mukteswar evolved into the velogenic strain JS/7/05/Ch with six amino acid mutations in the HN protein. We previously identified the HN mutant as having a crucial role in increasing the virulence of Mukteswar [26]. However, the host molecules involved in NDV infection remain unclear.

Further exploration is needed to investigate the host molecules involved in the infection process of genotype III NDVs. The present study aims to explore the effects of mutations on the interactions of the HN protein with host proteins and further investigate the impact of host molecules on NDV infection. Here, through LC‒MS/MS analysis, we successfully identified a host cellular protein, vimentin, which exhibited differential interactions with the prototypic and mutant HN proteins. Briefly, vimentin exhibited a robust interaction with the prototypic HN protein (Mukteswar-type HN) but not with the mutant HN protein (JS/7/05/Ch-type HN). This finding implied that the mutations in HN impaired the interaction between vimentin and HN. As an important intermediate filament protein, vimentin is widely involved in the infectious process of various viruses, including DNA, single-stranded RNA and double-stranded RNA viruses [7]. Viral infection can also trigger vimentin rearrangement, which contributes to viral replication [30]. The velogenic variant strain JS/7/05/Ch mediated a more significant rearrangement of vimentin fibres due to its mutant HN. Furthermore, vimentin rearrangement and NDV replication were reciprocally dependent. In essence, vimentin rearrangement proved to be a crucial event facilitating NDV replication, which was particularly evident in the case of the HN mutant strain JS/7/05/Ch. Conversely, efficient NDV replication hinged upon successful vimentin rearrangement. This interesting finding can also be observed in infections with other viruses, such as PRRSV [30] and FMDV [24]. Therefore, the significant rearrangement of vimentin induced by the velogenic variant NDV strain plays a key role in viral replication. Moreover, vimentin functions as a double-edged sword in virus replication. On the one hand, it facilitates the replication of some viruses, such as AIV [22] and FMDV [19]; on the other hand, it can be an inhibitor of viral replication, as seen for viruses such as HIPV3 [35] and HIV-1 [25]. Here, we further investigated the role of vimentin in NDV replication. Our findings indicated that vimentin was required for NDV replication and exerted a greater effect on the replication of the velogenic variant NDV strain due to its mutant HN. Based on the above results, we speculate that the strong interaction between prototypic HN and vimentin weakens the reciprocal dependence between NDV infection and vimentin rearrangement. Of note, the HN mutant has been identified as the key factor in the virulence of genotype III NDVs after intravenous infection, and mononuclear macrophages have been identified as the susceptible target cells of genotype III NDVs in peripheral blood mononuclear cells [26, 27]. Therefore, we conducted comparative experiments between HD11 chicken macrophages and CEFs. The comparative results revealed that the differential interactions between NDV infection and vimentin were pronounced in HD11 cells but not in CEFs. Moreover, the rearranged vimentin filaments and viral dsRNA exhibited widespread colocalization in HD11 cells. However, this phenomenon was not observed in CEFs, as shown in Figure 4. This suggests that the rearranged vimentin filaments can act as a replication factory for the variant NDV in chicken macrophages. These findings also provide further evidence of the underlying impact of vimentin on the variable virulence of genotype III NDVs upon intravenous infection.

Vimentin has been demonstrated to play a key role in numerous stages of viral infection [7]. To elucidate the specific role of vimentin during NDV infection, we further assessed the levels of viral attachment, internalization, fusion, and release in HD11 cells. Viral attachment is crucial for the successful establishment of viral infection [33]. Vimentin can play a dual role in viral infection by serving as a coreceptor for SARS-CoV [8] and SARS-CoV-2 [9] to enhance viral attachment, while it can also impede the attachment of AIV by disrupting the formation of lipid rafts on the surface of host cells [36]. In this study, cell surface vimentin had no significant effect on the attachment of genotype III NDVs. This interesting finding suggests that vimentin may not function as a coreceptor during NDV infection but instead may influence the infection process following viral attachment. Therefore, we conducted further assays to investigate the role of vimentin in the subsequent infection processes of genotype III NDV. Internalization is the first step of viral entry, facilitating the transport of virus particles into the cytoplasm [37]. Our results showed that vimentin was required for internalization of the velogenic variant NDV strain but not the mesogenic strain. Fusion between viral and cellular membranes is a crucial step in the infection process of enveloped viruses [38]. Herein, vimentin had a more significant impact on the fusion of the velogenic variant NDV strain than on that of the mesogenic strain. Generally, the release of virions can be considered to be the last stage of viral infection [39]. The findings of this study showed that vimentin significantly influenced the release of the velogenic variant NDV strain compared to the mesogenic strain. Taken together, these results indicate that vimentin plays an important role in later infection processes following NDV attachment. Moreover, the varied impacts of vimentin on viral infection eventually lead to differences in NDV replication efficiency. Notably, the robust interaction between the prototypic HN and vimentin may weaken the impact of vimentin on these infection processes. However, there are still certain limitations to this study. For instance, what is the impact of vimentin on the infection processes of other NDV genotypes? What is the precise underlying mechanism governing the contrasting interactions between vimentin and HN proteins during viral infection? These questions require additional elucidation in forthcoming research endeavours.

In summary, this study successfully confirmed that vimentin is associated with NDV infection. The velogenic variant NDV strain exhibited an advantage over the mesogenic strain in inducing the rearrangement of vimentin. Moreover, the efficient replication of the variant strain was strongly dependent upon vimentin rearrangement. Notably, the differential interaction between NDV infection and vimentin rearrangement was pronounced in HD11 cells. In addition, vimentin exerted more significant effects on multiple infection processes of the velogenic variant NDV strain. These findings provide further evidence supporting the underlying role of vimentin in the differences in the virulence of genotype III NDVs.

### Supplementary Information

**Additional file 1. Base peaks in NDV-infected samples.**

**Additional file 2. Peptide and protein identification by LC‒MS/MS**

**Additional file 3. Immunofluorescence assay showing the differential impacts of vimentin knockdown and overexpression on NDV replication.** HD11 cells in 12-well cell culture plates were transfected with siRNA (siRNA-VIM or siRNA-NC) or an expression plasmid (pcDNA3.1-VIM or pcDNA3.1). After transfection, the cells were infected with JS/7/05/Ch, JS/MukHN, or Mukteswar at an MOI of 1 for 24 h. The infected cells were fixed with cold 4% paraformaldehyde, permeabilized with Triton X-100, and blocked with 5% BSA. Vimentin (red) and NDV NP (green) were subsequently visualized using specific antibodies on a fluorescence microscope, with the nuclei stained with DAPI (blue). The mock-infected cells were assayed in parallel as a control. Images were captured at 200× magnification. Scale bars, 100 μm.

**Additional file 4. Validation of the efficacy of the anti-vimentin antibody in blocking cell surface vimentin**. HD11 cells in 6-well cell culture plates were pretreated with an anti-vimentin antibody and then exposed to JS/7/05/Ch, JS/MukHN, or Mukteswar at an MOI of 1. The infected cells were harvested and lysed at 24 hpi, and the assay was conducted according to the manufacturer's instructions for the Co-Immunoprecipitation Kit (Santa Cruz Biotechnology, USA). The immunoprecipitated proteins were then identified and analysed by Western blotting using an anti-HN antibody, an anti-vimentin antibody or normal mouse IgG. The band greyscale values were determined by ImageJ 1.4 software. The ratio of the vimentin protein level to the HN protein level represents the HN-vimentin interaction level. Three groups of cells were included in the co-IP experiment: the input group, antibody group and IgG group.

## Data Availability

The data that support the findings of this study are available on request from the corresponding author (xfliu@yzu.edu.cn).
