# Supplementary figures and images for: Cellular vimentin regulates the infectivity of Newcastle disease virus through targeting of the HN protein
Source: Vet Res. 2023 Oct 17;54:92. doi: 10.1186/s13567-023-01230-5 (PMC10580610; doi:10.1186/s13567-023-01230-5)

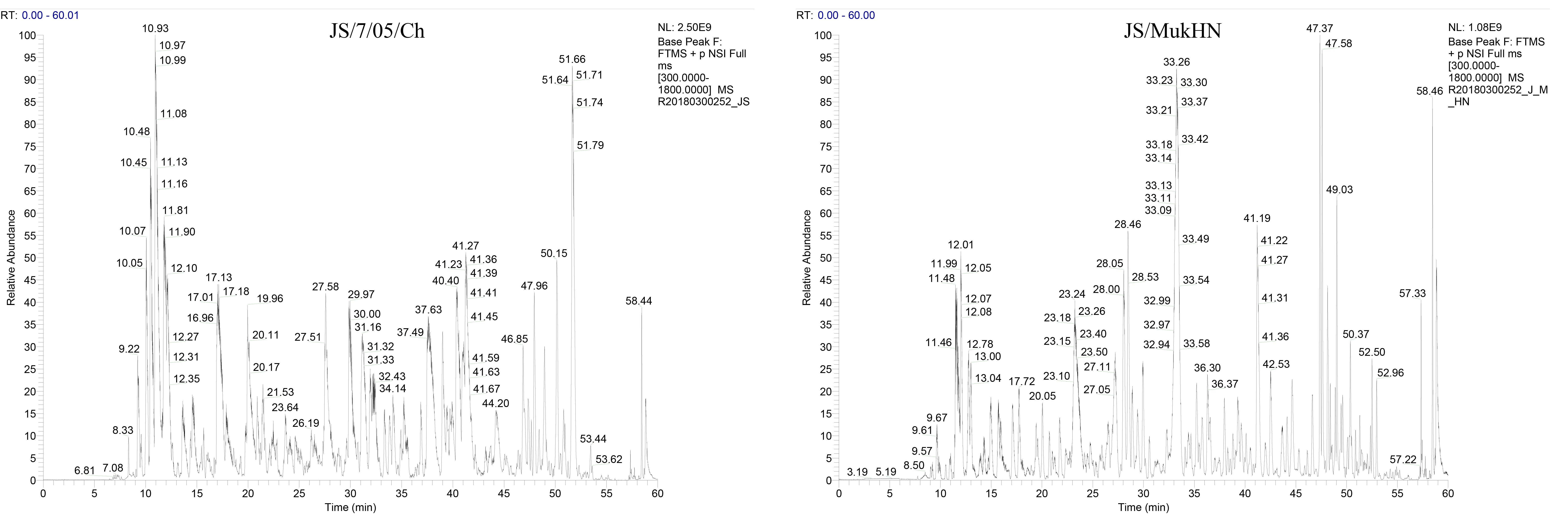

Supplement: Supplementary file 1 — Additional file 1. Base peaks in NDV-infected samples. [file 13567_2023_1230_MOESM1_ESM.tif]

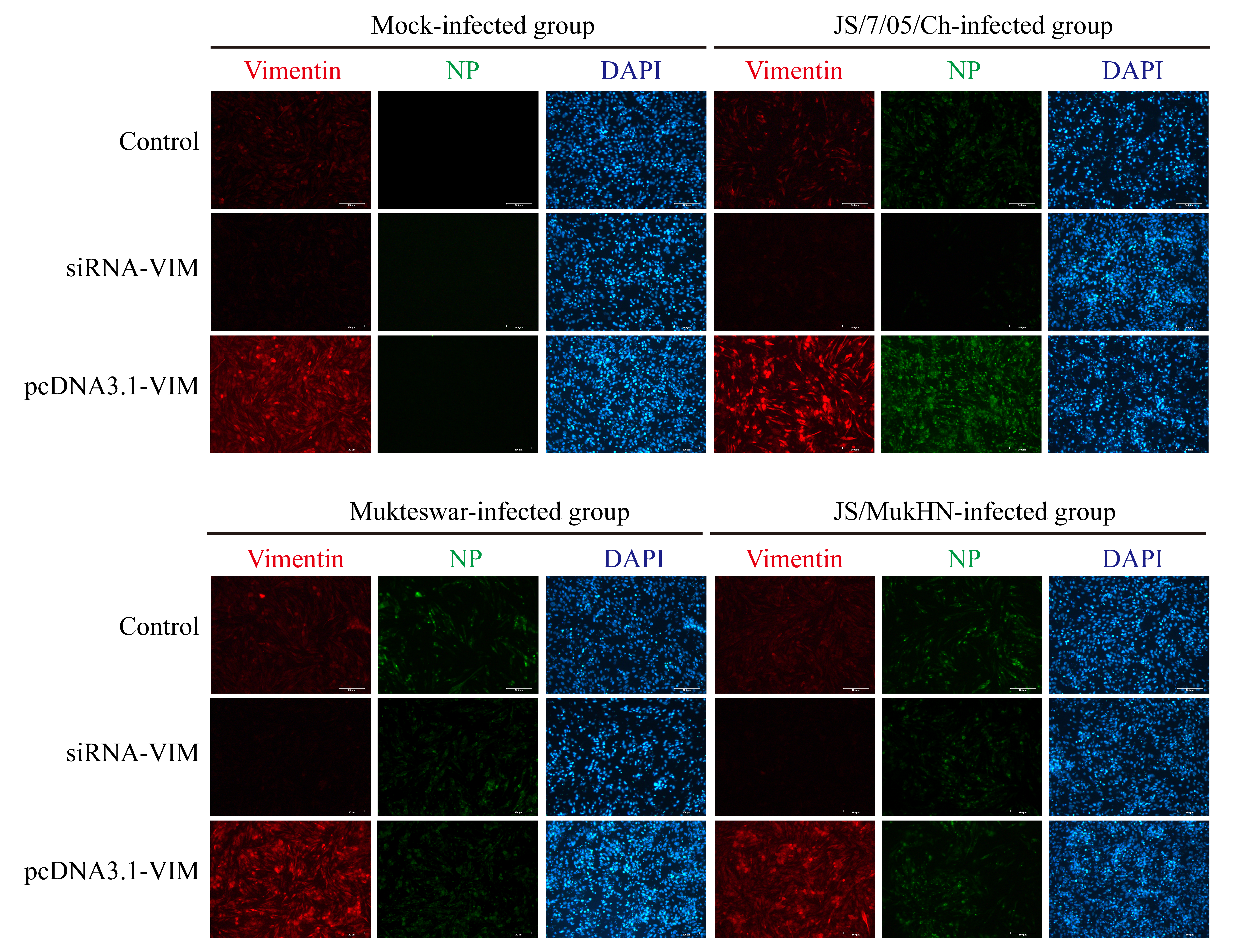

Supplement: Supplementary file 3 — Additional file 3. Immunofluorescence assay showing the differential impacts of vimentin knockdown and overexpression on NDV replication. HD11 cells in 12-well cell culture plates were transfected with siRNA (siRNA-VIM or siRNA-NC) or an expression plasmid (pcDNA3.1-VIM or pcDNA3.1). After transfection, the cells were infected with JS/7/05/Ch, JS/MukHN, or Mukteswar at an MOI of 1 for 24 h. The infected cells were fixed with cold 4% paraformaldehyde, permeabilized with Triton X-100, and blocked with 5% BSA. Vimentin (red) and NDV NP (green) were subsequently visualized using specific antibodies on a fluorescence microscope, with the nuclei stained with DAPI (blue). The mock-infected cells were assayed in parallel as a control. Images were captured at 200× magnification. Scale bars, 100 μm. [file 13567_2023_1230_MOESM3_ESM.tif]

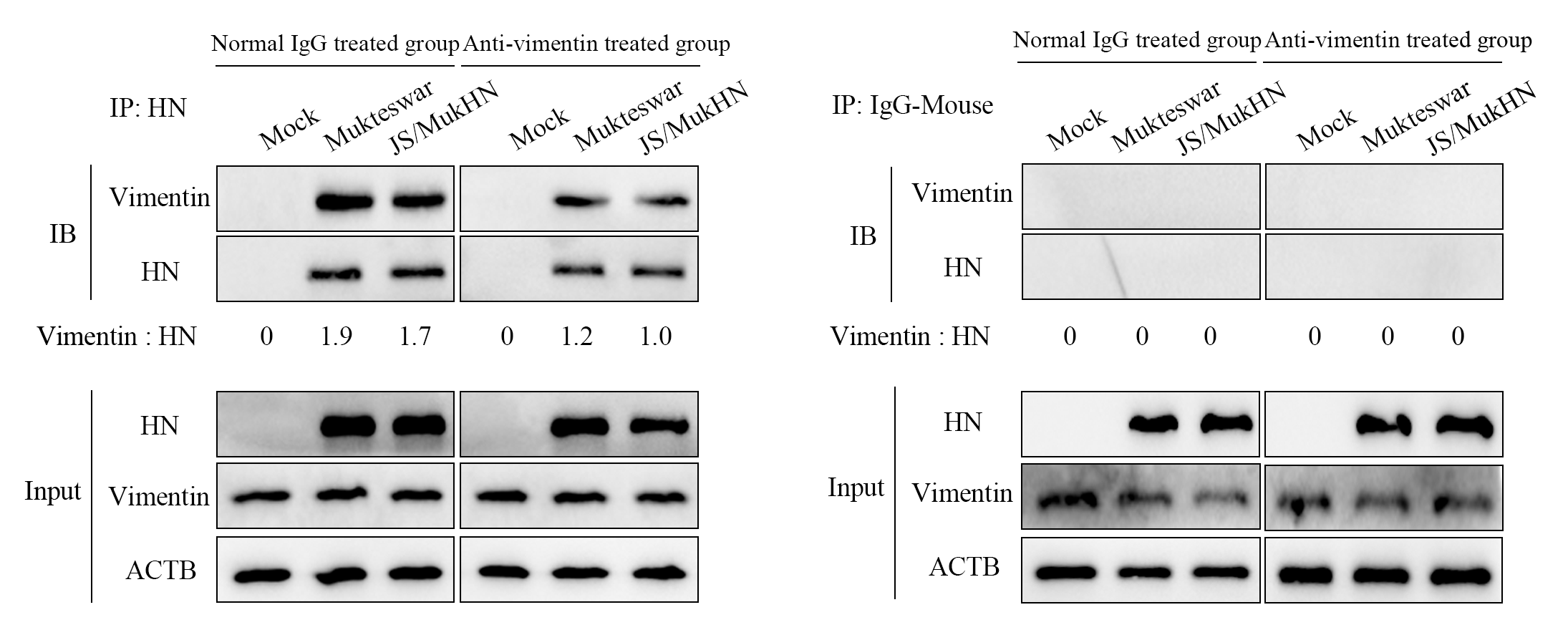

Supplement: Supplementary file 4 — Additional file 4. Validation of the efficacy of the anti-vimentin antibody in blocking cell surface vimentin. HD11 cells in 6-well cell culture plates were pretreated with an anti-vimentin antibody and then exposed to JS/7/05/Ch, JS/MukHN, or Mukteswar at an MOI of 1. The infected cells were harvested and lysed at 24 hpi, and the assay was conducted according to the manufacturer's instructions for the Co-Immunoprecipitation Kit (Santa Cruz Biotechnology, USA). The immunoprecipitated proteins were then identified and analysed by Western blotting using an anti-HN antibody, an anti-vimentin antibody or normal mouse IgG. The band greyscale values were determined by ImageJ 1.4 software. The ratio of the vimentin protein level to the HN protein level represents the HN-vimentin interaction level. Three groups of cells were included in the co-IP experiment: the input group, antibody group and IgG group. [file 13567_2023_1230_MOESM4_ESM.tif]
